# Antioxidant Effect of Octopus Byproducts in Canned Horse Mackerel (*Trachurus trachurus*) Previously Subjected to Different Frozen Storage Times

**DOI:** 10.3390/antiox11112091

**Published:** 2022-10-23

**Authors:** Lucía Méndez, Marcos Trigo, Bin Zhang, Santiago P. Aubourg

**Affiliations:** 1 Department of Food Technology, Marine Research Institute (CSIC), c/Eduardo Cabello, 6. 36208 Vigo, Spain; 2 Key Laboratory of Health Risk Factors for Seafood of Zhejiang Province, College of Food Science and Pharmacy, Zhejiang Ocean University, Zhoushan 316022, China

**Keywords:** horse mackerel, frozen storage, canning, octopus processing, cooking liquor, packing medium, lipids, oxidation, hydrolysis, colour

## Abstract

The effects on lipid damage in canned horse mackerel (*Trachurus trachurus*) of a prior frozen storage (−18 °C) period and the presence of an octopus (*Octopus vulgaris*) cooking juice (OCJ) in the packing medium were investigated. An increase of the frozen storage time favoured an increase (*p* < 0.05) of free fatty acid (FFA), peroxide, and thiobarbituric acid reactive substance contents and a decrease (*p* < 0.05) of the phospholipid (PL) value and polyene index. Furthermore, an increased presence of OCJ in the packing medium led to an inhibitory effect (*p* < 0.05) on fluorescent compound formation as well as to a retention (*p* < 0.05) of the PL and FFA compounds. Colour determination showed a substantial increase (*p* < 0.05) of L* and b* values in canned fish with previous frozen storage time. Nevertheless, this increase was partly reduced (*p* < 0.05) by the OCJ presence in the packing medium. It is concluded that previous holding time has led to an increased lipid oxidation development and loss of beneficial lipid constituents (i.e., PLs and polyunsaturated fatty acids). Remarkably, the presence in the packing medium of preservative compounds (i.e., antioxidants) included in waste juice obtained from octopus processing provided an effective tool for lipid preservation and quality enhancement in canned fish.

## 1. Introduction

Among traditional technologies, canning represents one of the most important possibilities of fish preservation. In it, heat treatment involved can substantially alter the nature of the initial substrate and lead to a food product with different properties, which can support an important role in human nutrition [1,2]. However, since most species employed as raw material employed in canneries occur in glut quantities, producers need to hold the initial material before it is processed or just carried to the factory. For it, most of the quality problems concerning canned seafood can be related to the quality of the initial material, which continuously changes during the holding period [3,4]. As a cooling strategy, frozen storage has been the most employed method on the basis of partially inhibiting most damage mechanisms (i.e., lipid oxidation, endogenous enzyme activity, and microbial decomposition) and retaining fish quality for a reasonable time [5,6]. However, if relatively long periods are required during the previous frozen storage or if the holding temperature is not correctly kept during the distribution chain, lipid hydrolysis and oxidation have shown to occur and to influence fish acceptance [7,8].

In order to increase the rancidity stability of canned seafood, natural antioxidants from different natural sources have been tested as packing systems. Thus, the high presence of phenolic compounds in extra-virgin olive oil has proved a marked inhibition of lipid oxidation development in canned tuna (*Thunnus alalunga*) when compared to brine-canned fish [9]. Similarly, a lower hydrolytic and oxidative damage was detected in canned fish containing extra-virgin olive oil as packing medium when compared to other kinds of oils [10]. Later on, the employment of sunflower oil as packing medium led to a lower hydrolysis and oxidation development in canned yellowfin tuna (*Thunnus albacares*) when compared to other coating oils [11]. An inhibitory effect on lipid oxidation development was proved by inclusion of macroalgae extracts both in water-packed (*Bifurcaria bifurcata)* [12] and brine-packed (*Fucus spiralis* and *Ulva lactuca*) [13] canned fish. Additionally, the introduction of alga *Fucus spiralis* extracts in the packing medium led to an increased rancidity stability in canned Chub mackerel (*Scomber colias*) that was previously stored (0–9-day period) under chilling conditions [14].

Octopus species constitute highly nutritional seafood that are commercialised in a great variety of products. Among the different wastes resulting from octopus processing, cooking juice or liquor has attracted a great interest from technologists and fish trade on the basis of its preserving and healthy properties. Thus, Oh et al. [15] detected remarkable antioxidant and antihypertensive effects, while Kim et al. [16] proved that cooking drip resulting from Giant Pacific octopus (*Enteroctopus dofleini*) processing showed a radical scavenging activity and an inhibitory activity against tyrosine and angiotensin I converting enzyme. On the basis of the DPPH assay, an antioxidant capacity was proved in cooking drip from the same octopus species by Choi et al. [17]; notably, this effect showed to increase by employing a previous gamma-irradiation treatment. Recently, the introduction of octopus (*Octopus vulgaris*) cooking liquor as packing system led to an increased rancidity stability of water-canned Chub mackerel (*Scomber colias*) [18].

In the present study, the effects of a prior frozen storage period (0–6-month period) and the presence of cooking juice of common octopus (*Octopus vulgaris*) in the packing medium were investigated in brine-canned horse mackerel (*Trachurus trachurus*). For it, quality analyses related to lipid damage (lipid hydrolysis and oxidation; phospholipid and polyene index values) and muscle colour (L*, a*, and b* parameters) were carried out in initial and canned horse mackerel.

## 2. Materials and Methods

### 2.1. Octopus Cooking Juice, Initial Fish and Fish Frozen Storage

Octopus (*O. vulgaris*) cooking juice (OCJ) was facilitated by *Frigoríficos Rosa de los Vientos S. L.* (Marín, Pontevedra, Spain). For it, 2 L of commercial vacuum-sealed juice were employed. The product was stored under refrigerated conditions (4 °C) before employment.

**Figure 1:**
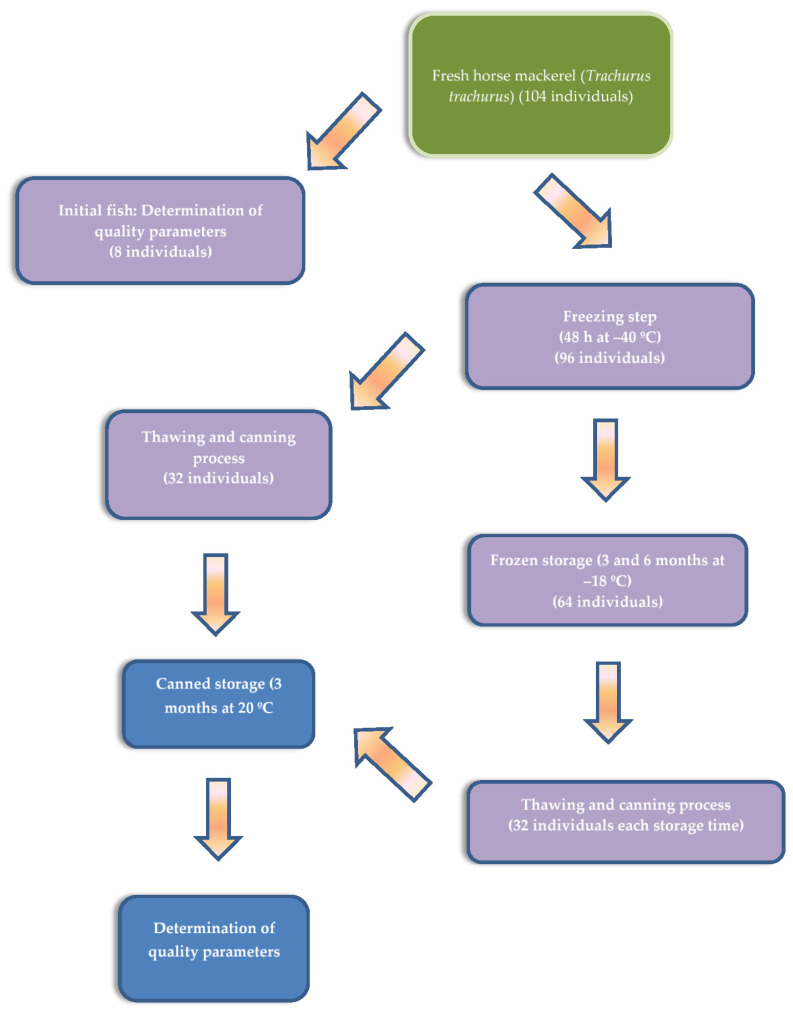
Summarised steps carried out in the present experimental procedure.

Specimens (104 fish) of fresh horse mackerel (*T. trachurus*) (length and weight ranges: 25.5–29.0 cm and 163–179 g, respectively) were obtained at Vigo harbour (northwestern Spain) and transported on ice to the laboratory within 20 min. The different steps carried out in the present experimental procedure are expressed in Figure 1. As a first step, 8 fish specimens were separated and divided into four groups (two specimens per group). The fish (raw or initial fish) were beheaded, eviscerated, and filleted. Then, the white muscle of the fillets was separated, pooled together within each group, minced, and analysed independently (*n* = 4).

The remaining fish individuals (96 fish) were stored at −40 °C for 48 h. After this time, 32 fish individuals were thawed overnight (4 °C) and then subjected to the canning process (canned fish corresponding to the 0-month frozen period). In the meantime, 64 (two 32 individual groups) fish were kept frozen (−18 °C) for 3 and 6 months, respectively (canned fish corresponding to the 3-month and 6-month frozen periods). At each sampling time, specimens (32 fish) were thawed overnight (4 °C) and then subjected to the canning process.

### 2.2. Canning Process

Thawed specimens were beheaded, eviscerated, and filleted. Then, 45 g portions of fish fillets were taken and placed in small flat rectangular cans (105 × 60 × 25 mm; 150 mL). As packing media, 0, 5, 15, and 30 mL of OCJ were introduced in the cans, followed by the addition of 40, 35, 25, and 10 mL of distilled water, respectively. Then, 40 mL of aq. 4% NaCl solution were added to each can. Packing conditions prepared were named as control (CT batch), low-concentrated (OCJ-1 batch), medium-concentrated (OCJ-2 batch), and high-concentrated (OCJ-3 batch), respectively. Each can was prepared with a single fish.

All cans were vacuum-sealed and then subjected to sterilisation in a horizontal steam retort (115 °C, 45 min; *F*_o_ = 7 min) (CIFP Coroso, Ribeira, A Coruña, Spain). After finishing the heating time, steam was cut off, and air was used to flush away the remaining steam. The cans were cooled at a reduced pressure. Cans were stored at room temperature (20 °C) for 3 months. After this time, the cans were opened, the liquid part was carefully drained off gravimetrically, and the content of the can was filtered through a filter paper. The fish white muscle was considered for the study; for it, it was separated, wrapped in filter paper, and used for analysis.

At each sampling time of the canned fish, the white muscle of two cans with the same OCJ content was pooled together, minced, and employed to carry out the different quality analyses. Each batch (CT, OCJ-1, OCJ-2, and OCJ-3) was analysed by means of four replicates (*n* = 4).

A brine filling medium (i.e., final aq. 2% NaCl concentration) was employed in the present study as being a common commercial filling medium employed for fish canning. Additionally, such hydrophilic packing medium would prevent possible interactions of the fish muscle lipids with a filling medium including a lipid component (i.e., vegetable oil).

The choice of the OCJ content tested in this canning study was based on preliminary tests. Thus, a 30 mL volume addition (i.e., OCJ-3 batch) showed to correspond to the highest concentration possible without modifying the sensory descriptors of the canned horse mackerel (i.e., flesh colour, odour, or flavour) under the current brine-packing conditions. Additionally, two lower volumes (namely, 5 and 15 mL, OCJ-1 and OCJ-2 batches, respectively) were also checked in this study to analyse the effect of the OCJ content.

Chemical reagents and solvents used in this study were of reagent grade (Merck, Darmstadt, Germany).

### 2.3. Moisture Determination and Lipid Extraction and Quantification in Initial Fish and Canned Fish

Determination of moisture in fish muscle was carried out as the weight difference (1–2 g) before and after heating for 4 h at 105 °C according to the official method 950.46 B [19]. Results were calculated as g·kg^−1^ fish muscle.

Extraction of lipids of fish muscle was done following the Bligh and Dyer [20] method, which employs a single-phase solubilisation of the lipids using a chloroform-methanol (1:1) mixture. Quantification was undergone according to Herbes and Allen [21]. Results were calculated as g·kg^−1^ fish muscle.

### 2.4. Determination of Rancidity Stability in Initial Fish and Canned Fish

Peroxide value (PV) was determined spectrophotometrically (520 nm; Beckman Coulter DU 640 spectrophotometer, Brea, CA, USA) according to the method developed by Chapman and McKay [22], in which peroxides included in the lipid fraction are reduced with ferric thiocyanate. Results were calculated as meq. active oxygen·kg^−1^ lipids.

Thiobarbituric acid index (TBA-i) was analysed according to the method proposed by Vyncke [23]. This method is based on the reaction between a trichloroacetic acid extract of the fish muscle and thiobarbituric acid. The content of thiobarbituric acid reactive substances (TBARS) was measured spectrophotometrically at 532 nm and calculated from a standard curve that was prepared using 1,1,3,3-tetraethoxy-propane (TEP); this curve was obtained from an aqueous solution of TEP (0.24 mL TEP·L^−1^) including a range of 0.1 × 10^−8^ to 4.0 × 10^−8^ moles of malondialdehyde. The results were calculated as mg malondialdehyde·kg^−1^ fish muscle.

Fluorescent compound formation (Fluorimeter LS 45; Perkin Elmer España; Tres Cantos, Madrid, Spain) was measured in the aqueous fraction obtained from the lipid extraction process of horse mackerel muscle [24]. For it, fluorescence development was measured at excitation/emission at 393/463 and 327/415 nm. The relative fluorescence (RF) was calculated according to the following formula: RF = *F/F_st_*, where *F* is the fluorescence measured at each excitation/emission wavelength pair, and *F_st_* is the fluorescence intensity of a quinine sulphate solution (1 µg·mL^−1^ in 0.05 M H_2_SO_4_) measured at the corresponding wavelength pair. The fluorescence ratio (FR) was calculated as the ratio between the two RF values: FR = RF_393/463 nm_/RF_327/415 nm_.

Lipid extracts were converted into fatty acid methyl esters (FAME) by using acetyl chloride in methanol. Then, they were analysed by gas–liquid chromatography (Perkin Elmer 8700 chromatograph, Madrid, Spain) [25]. Identification of peaks corresponding to FAME was carried out by comparison of their retention times with those of commercial standard mixtures (Qualmix Fish, Larodan, Malmo, Sweden; FAME Mix, Supelco, Inc., Bellefonte, PA, USA). For quantitative purposes, peak areas were automatically integrated, with C19:0 fatty acid being used as the internal standard. The content of each fatty acid (FA) was calculated as g·100 g^−1^ total FA. The polyene index (PI) was calculated as the following FA content ratio [25]: (C20:5ω3 + C22:6ω3)/C16:0.

### 2.5. Determination of Free Fatty Acid (FFA) and Phospholipid (PL) Contents in Initial Fish and Canned Fish

FFA content in the lipid extract was spectrophotometrically (710 nm) determined following the method of Lowry and Tinsley [26]; this method is based on the formation of a complex with cupric acetate–pyridine. Results were calculated as g FFA·kg^−1^ fish muscle and g FFA·kg^−1^ lipids.

PL content was measured according to the method of Raheja et al. [27]; this method is based on formation of a complex with ammonium molybdate. Results were calculated as g PL·kg^−1^ fish muscle and g PL·kg^−1^ lipids.

### 2.6. Colour Assessment in Initial Fish and Canned Fish

A tristimulus HunterLab Labscan 2.0/45 colorimeter (Reston, VA, USA) was employed to carry out the instrumental colour determination (CIE 1976). Colour parameter (L*, a*, and b*) scores corresponding to each muscle sample (initial and canned) were averaged over four measurements; such measurements were taken by rotating the measuring head 90° among triplicate measurements per position.

### 2.7. Statistical Analysis

Data (*n* = 4) obtained from the different physicochemical analyses were subjected to one-way ANOVA (*p* < 0.05) to investigate differences resulting from previous frozen storage time and from concentration of OCJ in the packing medium (Statistica version 6.0, 2001; Statsoft Inc., Tulsa, OK, USA). Comparison of means was carried out by using a least-squares difference (LSD) method.

## 3. Results and Discussion

### 3.1. Determination of Moisture and Lipid Contents

Moisture and lipid contents of initial horse mackerel were 773.9 ± 4.3 and 14.2 ± 2.4 g·kg^−1^ muscle, respectively. Contents for both constituents agree with previous studies related to this pelagic medium-fat species [25,28]. Canning process led to a substantial moisture loss (range of 740–755 g·kg^−1^ muscle), while the lipid content showed a marked increase (range of 17.5–22.5 g·kg^−1^ muscle). Notably, no effect (*p* > 0.05) on the content of both constituents in canned muscle could be detected as a result of previous frozen storage time and presence of OCJ in the packing medium (data not shown).

Moisture loss in the canned muscle can be explained as a result of protein denaturation during the sterilisation process, which in turn leads to a decrease of water-holding capacity of muscle [3,5,29]. As a result, a drip loss into the packing medium would be produced, so that other constituents such as the lipid fraction would increase their relative content in canned muscle, which agrees with current results on lipid content in canned horse mackerel.

Previous research related to the effect on moisture and lipid contents of canned fish muscle of previous frozen storage time and packing media, including preservative compounds, can be considered very scarce. Thus, no effect of previous frozen storage time (0–15-month period) of sardine (*Sardina pilchardus*) was detected on the water and lipid contents of the corresponding canned product [30]. According to the present study, previous research also employing the juice resulting from octopus (*O. vulgaris*) cooking did not provide differences for moisture and lipid contents in water-canned Atlantic mackerel (*S. scombrus*) [18]. No differences in lipid content were obtained by Barbosa et al. [13] in canned Chub mackerel (*S. colias*) when aqueous extracts of alga (*Fucus spiralis* or *Ulva lactuca*) were included in the filling medium.

### 3.2. Assessment of Lipid Oxidation Development

Determination of lipid oxidation was carried out by following different and complementary analytical tools, i.e., including indices related to primary, secondary, and tertiary lipid oxidation compound formation.

Peroxide levels were very low in initial fish (1.56 ± 0.71 meq·kg^−1^ lipids; Table 1) and showed a substantial increase after the canning process, with all canned values being included in the 1.53–6.66 meq·kg^−1^ lipids range. Additionally, an increasing tendency (*p* < 0.05) was detected in all batches by increasing the previous holding time. Thus, a significant increase (*p* < 0.05) was detected in all cases when comparing samples stored for 3 months with their counterparts from the 0-month period. Concerning the content of the OCJ in the packing medium, no significant differences could be detected (*p* > 0.05). It can be concluded that the presence of OCJ in the packing medium did not produce any effect on the peroxide content in canned fish.

Very low TBA-i values were detected in the initial fish (Table 1). However, a substantial increase was detected in the canned fish corresponding to the 0-month storage as a result of the sterilisation process. Additionally, a progressive TBA-i increase (*p* < 0.05) was observed in all batches by increasing the previous frozen storage time. However, values were in all cases included in the 0.13–0.54 mg malondialdehyde·kg^−1^ muscle range, so that a great formation of secondary lipid oxidation compounds could not be inferred [25,31]. Related to the OCJ presence in the packing medium, significant differences (*p* > 0.05) were very scarce so that a definite effect on the formation of secondary lipid oxidation compounds could not be concluded.

Detection of tertiary lipid oxidation compounds (i.e., FR value) was carried out by assessing the interaction compound formation between primary and secondary lipid oxidation compounds and nucleophilic compounds present in the fish muscle (i.e., protein-type molecules) [25,30]. Results showed a marked increase as a result of the canning process (Table 2), which can be explained by the effect of the heating step involved (i.e., sterilisation). This FR increase was even higher (*p* < 0.05) by increasing the previous holding time, with differences being significant (*p* < 0.05) for control canned fish previously stored for 3 months. In general, a progressive increase of average FR values was proved by increasing the previous storage period. The presence of OCJ in the packing medium did not lead to differences in samples corresponding to the 0-month storage. However, when considering canned samples previously stored for 3 months, all OCJ batches showed lower (*p* < 0.05) FR values than their canned control counterparts. A significantly lower (*p* < 0.05) FR value was also detected in canned horse mackerel corresponding to the OCJ-3 batch when compared to the canned control. It is concluded that the presence of the OCJ in the packing medium has led to an inhibitory effect on the formation of tertiary lipid oxidation compounds.

The lipid oxidation mechanism is considered a complex deteriorative pathway involving the formation of a great diversity of molecules. The general lipid oxidation development found in the present study can be explained on the basis of several factors. First, pro-oxidant endogenous enzymes (i.e., peroxidases, lipoxygenases, etc.) would catalyse this damage mechanism during the fish frozen storage [7,8]. Additionally, the strong heating treatment involved in the canning process would accelerate the formation of the different lipid oxidation compounds (i.e., primary, secondary, and tertiary) [2,3]. Finally, the presence in the packing medium of preservative compounds (namely, antioxidants) included in the OCJ would decrease this lipid oxidation development, this leading to a lower formation of oxidised lipid molecules (i.e., tertiary lipid oxidation compounds).

Previous research accounts for a wide number of studies showing the antioxidant properties of cooking juices resulting from the commercial processing of tuna species, this effect being attributed in most cases to lower-molecular-weight peptides [32,33]. Concerning the preservative properties of liquors resulting from octopus species cooking, several studies can be mentioned. Thus, Oh et al. [15] analysed the components of octopus cooking liquors and demonstrated an antioxidant behaviour on the basis of carrying out a Rancimat assay. In the same way, cooking liquor from Giant Pacific octopus (*E. dofleini*) revealed an antioxidant behaviour (DPPH assay) that could be increased if a previous gamma-irradiation process was applied [17]. Kim et al. [16] demonstrated that a 70% ethanol extract from the cooking juice of Giant Pacific octopus (*E. dofleini*) included a great content of polyphenol molecules; additionally, FRAP and DPPH assays proved a radical scavenging behaviour and inhibitory properties against tyrosine and angiotensin I-converting enzyme. Recently, an inhibitory effect on fluorescent compound formation was detected in water-canned Chub mackerel (*S. colias*) when including octopus cooking liquor in the packing medium [18].

Previous research also accounts for the antioxidant effect of other natural extracts included in the packing medium during fish canning. Thus, the employment of extra-virgin olive oil as a packing medium led to a marked inhibition of lipid oxidation development in canned tuna (*T. alalunga*) when compared to fish packed in brine solution [9]; this preservative effect was attributed to the high presence of polyphenol molecules in extra-virgin olive oil. In a subsequent study, polyphenols extracted from extra-virgin olive oil and included in the packing medium showed to be effective for inhibiting the lipid oxidation development of canned tuna (*T. alalunga*) [34]. Later on, Caponio et al. [10] showed the lowest hydrolytic and oxidative damage in canned fish (tuna, sardine, anchovy, mackerel) containing extra-virgin olive oil when compared to their counterpart canned fish packed under olive oil or refined seed oil. A lower formation of fluorescent compounds was detected by Naseri and Rezaei [35] in canned sprat (*Clupeonella cultriventris*) packed in sunflower oil when compared to its counterpart packed in brine solution; this inhibitory effect on lipid oxidation was attributed to the presence of antioxidant compounds in the oily medium. Later on, the employment of sunflower oil as packing medium led to a lower FFA formation and a lower TBARS content in canned yellowfin tuna (*T. albacares*) when compared to coconut and ground nut oils as coating media [11]. Moreover, in agreement with the present study, the lipid oxidation development during canning was partially inhibited by the presence of macroalgae extracts both in water- and brine-packed fish [12,13]. An inhibitory effect of fluorescent compound formation was also detected in canned Chub mackerel (*S. colias*) that was previously subjected to chilling storage (0–9-day period) by including an *F. spiralis* extract in the packing medium [14].

Closely related to lipid oxidation development, the polyene index has shown to afford complementary knowledge on the development of this damage mechanism. Thus, it provides information on the content increase or decrease of the polyunsaturated fatty acids as a result of fish canning or fish processing in general; additionally, this index has shown to be directly related to the nutritional value [25,36]. Results obtained are depicted in Table 2. A general decrease of the average value was found by comparison of the initial fish and samples corresponding to the 0-month holding period; differences were found significant (*p* < 0.05) in the case of the OCJ-1 batch. A progressive decrease (*p* < 0.05) of average values was detected in all kinds of canned samples by increasing the previous storage time. Thus, the lowest average values in CT, OCJ-1, and OCJ-2 batches were detected in canned fish corresponding to the 6-month holding time. No significant differences (*p* > 0.05) were detected as a result of the OCJ presence in the packing medium. Average values showed the lowest levels in the control batch in the case of considering canned samples corresponding to a previous storage time of 3 and 6 months. In canned samples corresponding to the longest holding time, the highest average value was obtained in canned horse mackerel, including the most concentrated OCJ packing medium. However, a significant (*p* > 0.05) effect on PUFA damage could not be concluded in the present study.

A preservative effect on the PI retention has been reported in previous related research as a result of the addition of antioxidant compounds to the packing medium. In a closely related study, the presence in the packing medium of a cooking liquor resulting from octopus (*O. vulgaris*) processing led to a preservative effect on the PI score in water-canned Chub mackerel (*S. colias*) [18]. Furthermore, a significant PI retention in canned Atlantic salmon (*Salmo salar*) muscle was proved when packed in a water medium including an ulte (basal part of alga *Durvillaea antarctica*) extract [37]; on the contrary, no differences in effect were detected when other algae (cochayuyo, frond of *D. antarctica*; *U. lactuca*; *Pyropia columbina*) extracts were employed as packing systems. Furthermore, higher PI values were obtained in canned Atlantic Chub mackerel (*S. colias*) when *F. spiralis* or *U. lactuca* extracts were included in the packing system [13]. According to the present results, no differences of the PI were detected in 3-year canned sprat (*C. cultriventris*) by comparison with brine and sunflower oil as packaging media [35].

### 3.3. Determination of FFA and PL Contents

**Figure 2:**
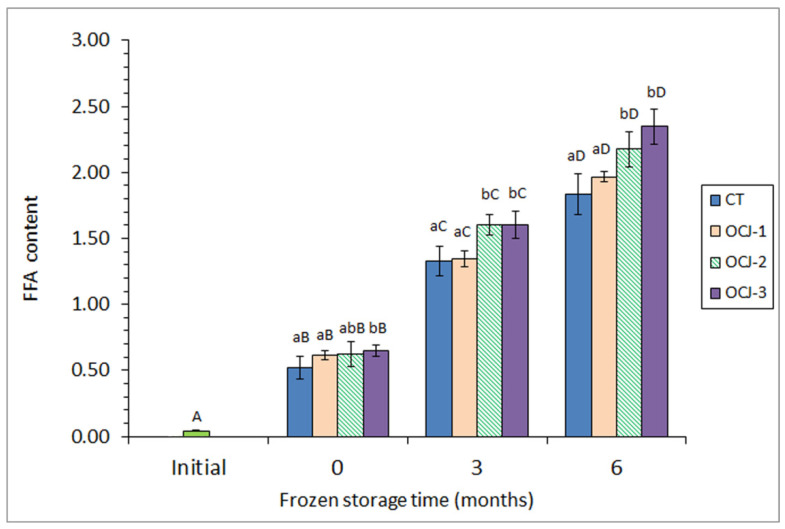
Determination of free fatty acid (FFA) content (g·kg^−1^ muscle) in initial and canned horse mackerel previously subjected to frozen storage and canned with different packing systems. Average values of four (*n* = 4) replicates; standard deviations are indicated by bars. Different capital letters (A–D) indicate significant differences (*p* < 0.05) as a result of previous frozen storage time. Different lowercase letters (a, b) indicate significant differences (*p* < 0.05) as a result of OCJ presence. Packing systems as expressed in Table 1.

A low FFA content was observed in initial fish (42.60 ± 3.32 mg·kg^−1^ muscle; Figure 2) (3.04 ± 0.52 g·kg^−1^ lipids), which corresponds to a high-quality fish substrate [25]. Comparison between initial fish and canned fish corresponding to the 0-month frozen period showed a marked increase (*p* < 0.05) of FFA content in fish muscle, which can be explained as a result of the thermal breakdown of higher-molecular weight lipid classes such as triacylglycerol and PL molecules. This FFA content increase showed to be more important (*p* < 0.05) by increasing the previous frozen storage time of the fish to be canned. Thus, the highest (*p* < 0.05) FFA levels were detected in canned fish previously subjected to a 6-month storage.

Remarkably, an increasing presence of OCJ in the packing medium led to an increased value of FFA content. Thus, canned samples corresponding to the OCJ-3 batch showed higher (*p* < 0.05) FFA contents than their counterparts from CT and OCJ-1 batches in all cases. For samples from the OCJ-2 batch, differences with the control batch were found significant (*p* < 0.05) when considering canned fish corresponding to 3 and 6 months of previous storage.

Results obtained for FFA levels can be influenced by several and opposite factors. One side, endogenous enzyme activity during the frozen storage period, would increase the FFA content, with this increase being more important with storage time [7,8]. On the other, the sterilisation process should breakdown higher-molecular weight lipid molecules and lead to a content increase of FFA values [24,30]. However, it has been proven that FFA compounds are more prone to undergo lipid oxidation development than higher-molecular weight molecules [38,39]. Therefore, the oxidation of FFA ought to be produced during the frozen storage and especially during the sterilisation process, leading to an FFA content decrease. Finally, the presence of preservative molecules (i.e., antioxidants) in the OCJ can inhibit the lipid oxidation development so that a preservative effect on FFA compounds may be produced. On the basis of the results obtained, this preservative effect has been proven to take place, so that an increased retention of FFA molecules was detected in the present research by increasing the OCJ concentration in the packing medium.

Previous research provides contradictory results when addressing the effect of packing conditions on lipid hydrolysis development. According to the present results, higher average FFA contents were observed in canned Atlantic mackerel (*Scomber scombrus*) by including *B. bifurcata* aqueous extracts in the packing medium [12]. Additionally, higher average FFA values were observed in canned Chub mackerel (*S. colias*) by the presence of *F. spiralis* or *U. lactuca* extracts in the covering medium [13]. Contrary to the current results, the FFA values of canned Chub mackerel (*S. colias*) did not show differences as a result of employing aqueous extracts of macroalga *F. spiralis* as a packing medium [14]. In a closely related study, no effect on the FFA content in water-canned fish (*S. colias*) was detected by including octopus (*O. vulgaris*) cooking liquor as a packing medium [18].

**Figure 3:**
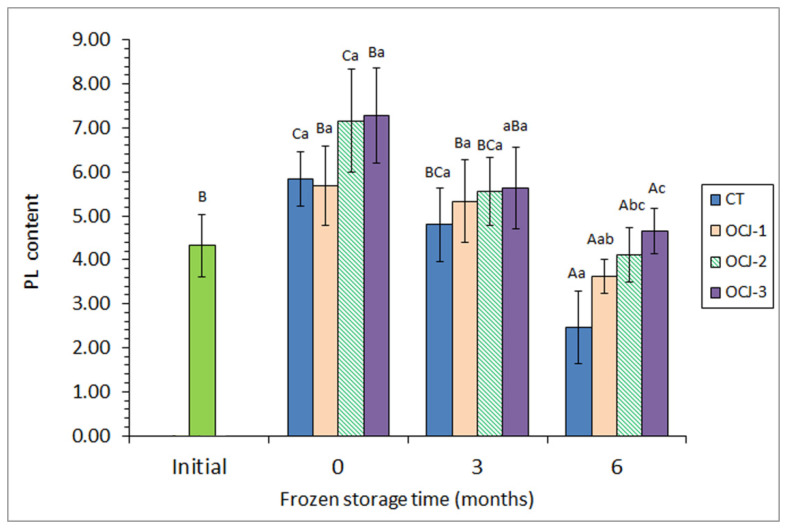
Determination of phospholipid (PL) content (g·kg^−1^ muscle) in initial and canned horse mackerel previously subjected to frozen storage and canned with different packing systems. Average values of four (*n* = 4) replicates; standard deviations are indicated by bars. Capital letters (A–C) indicate significant differences (*p* < 0.05) as a result of previous frozen storage time. Lowercase letters (a–c) indicate significant differences (*p* < 0.05) as a result of OCJ presence. Packing systems as expressed in Table 1.

The PL content of initial fish was 4.34 ± 0.71 g·kg^−1^ muscle (Figure 3) (305.51 ± 50.12 g·kg^−1^ lipids). Comparison between the initial fish and canned samples corresponding to the 0-month storage showed a general PL content increase. This increase can be explained as a result of marked losses on other constituents during the canning process such as moisture and hydrophilic constituents in general. Remarkably, an important decrease of the PL content in canned muscle was detected by increasing the previous frozen storage time; this tendency was observed in all batches and can be explained on the basis of the strong activity of endogenous enzymes (i.e., phopholipases) during the holding period [7,8]. A marked effect of OCJ presence in the packing medium was detected on PL content of canned fish, so that an increasing OCJ presence led to a higher average PL content. Canned samples corresponding to the 6-month storage revealed higher PL values in fish corresponding to OCJ-2 and OCJ-3 batches when compared to canned control fish. According to the results obtained, a preservative effect on the PL fraction is concluded for the OCJ when included in the packing medium.

PL compounds have been described as serving as drug delivery systems and having a high bioavailability and protecting effect on different kinds of diseases [40,41]. On the basis of pharmaceutical and food production industries, great efforts are being focused on the retention of PL compounds from marine species and their commercial byproducts as supporting a high polyunsaturated fatty acid content [31,42]. However, previous research concerning the effect of preservative compounds included in the packing medium on the PL content in canned fish muscle can be considered very scarce. According to the present results, an increased PL content was detected in canned Chub mackerel (*S. colias*) by including aqueous extracts of algae *F. spiralis* and *U. lactuca* in the packing medium [13]; this effect was found to increase with the alga concentration present in the packing medium.

### 3.4. Determination of Colour Changes

Assessment of average L* values revealed a marked increase with canning by comparison of initial fish and canned samples corresponding to the 0-month storage (Table 3); differences were found significant (*p* < 0.05) in all batches except for OCJ-1 fish. This increase agrees with previous research, showing an important lightness increase as a result of the canning process [3]. Furthermore, an increasing tendency (*p* < 0.05) of the L* value was detected in the current study with the previous holding time in all kinds of canned samples. Thus, the highest average values were obtained in canned fish corresponding to 6 months of frozen storage in all batches.

Remarkably, the presence of the OCJ in the packing medium led to an inhibitory effect on the L* value increase. Thus, canned fish corresponding to the two highest OCJ concentrations (i.e., OCJ-2 and OCJ-3 batches) showed lower (*p* < 0.05) values than fish corresponding to the control batch in all cases. It is concluded that OCJ presence has led to an inhibitory effect on the lightness increase in the canned fish. Determination of the a* colour value did not provide valuable results (data not shown), so that a definite effect of previous frozen storage time or presence of OCJ in the packing medium could not be inferred for this colour parameter.

Related to the b* value, on the basis of the comparison between initial fish and 0-month canned samples, a marked increase (*p* < 0.05) resulted from the canning process (Table 3). This increase agrees with previous research [43,44] and can be explained on the basis of the effect of the sterilisation step, which facilitates the interaction compound formation between oxidised lipids and protein-type molecules, and therefore the yellowish colour development. A progressive increase (*p* < 0.05) of average b* values was detected by increasing the previous holding time in all batches; as a result, the highest average values were detected in all cases in samples corresponding to the 6-month period. Concerning the presence of the OCJ, an inhibitory effect on the b* value increase was detected in canned fish corresponding to OCJ-3 batch (months 3 and 6) and OCJ-2 batch (month 6) when compared to the control batch. It is concluded that OCJ presence in the packing medium has led to an inhibitory effect on the yellowish colour development in canned fish, this measurement being closely related to lipid oxidation development [3,43].

Previous studies related to the effect of previous storage and packing conditions on the colour parameters of canned fish can be considered as scarce. Thus, increasing the previous storage time and temperature provoked an increase in the L* value and a decrease in the a* value in canned skipjack tuna (*Katsuwonus pelamis*) [45]. Conversely, previous chilling time (0–9–day period) did not lead to a significant effect on colour parameter values in canned and Coho salmon (*Oncorhynchus kisutch*) [24]. The employment of sunflower oil as packing medium led to lower L* and b* values in canned yellowfin tuna (*T. albacares*) when compared to coconut and ground nut oils as coating media [11]. Later on, a marked increase for L* and b* values in canned Atlantic mackerel (*S. scombrus*) by comparison with the initial raw fish was detected [12]; remarkably, this increase was partially inhibited by increasing the presence in the packing system of an aqueous extract of macroalga *B. bifurcata*. Recently, an inhibitory effect on the L* value increase was detected in canned Chub mackerel (*S. colias*) by including an *F. spiralis* aqueous extract in the packing medium [14]; additionally, a higher retention of the a* value was inferred by the alga extract presence in the coating system.

## 4. Conclusions

Previous frozen storage time and the presence of OCJ in the packing medium showed a substantial effect on the lipid quality and colour development in canned horse mackerel (*T. trachurus*). It is concluded that the previous holding time has led to an increased lipid damage development (oxidation and hydrolysis) and loss of beneficial lipid constituents (i.e., PLs and polyunsaturated fatty acids). However, the presence in the packing medium of preservative compounds (i.e., antioxidants) included in the waste juice obtained from octopus processing provided an effective tool for quality retention in canned fish according to lipid damage (FFA, PL, and FR) and external colour (L* and b*) determinations.

Food technology research is currently addressing an increasing attention to natural preservative strategies for the production of high-quality seafood and food in general. Furthermore, a great interest is also accorded to technologies susceptible to use commercial waste substrates that allow and facilitate the environmental sustainability and the circular economy. The current study provides a beneficial strategy consisting of the employment of a waste material to enhance the quality of commercial canned fish. This green strategy also agrees with present global interests in the search for effective antioxidants obtained from natural sources in order to replace synthetic antioxidants in food in general. On the basis of the relevance of the results obtained in the present research, further studies are envisaged to optimise and scale-up the employment of OCJ in different kinds of canned fish species.

## Figures and Tables

**Table 1:** Determination * of primary and secondary lipid oxidation in initial and canned horse mackerel previously subjected to frozen storage and canned with different packing systems **.

	Storage Time (Months)	Packing System			
		**CT**	**OCJ-1**	**OCJ-2**	**OCJ-3**
**PV** (meq·kg^−1^ lipids)	Initial	1.56 A (0.71)	1.56 A (0.71)	1.56 A (0.71)	1.56A (0.71)
	0	1.63 aA (0.42)	1.53 aA (0.22)	2.25 aA (0.51)	1.88 aA (0.25)
	3	4.43 aB (1.12)	6.22 aB (1.45)	5.02 aB (1.26)	5.39 aB (0.92)
	6	6.11 abC (1.82)	4.56 aB (1.30)	5.80 abB (1.70)	6.66 bB (0.76)
					
**TBA-i** (mg malondial-dehyde·kg^−1^ muscle)	Initial	0.06 A (0.03)	0.06 A (0.03)	0.06 A (0.03)	0.06 A (0.03)
	0	0.14 aAB (0.10)	0.24 aBC (0.16)	0.13 aB (0.06)	0.18 aB (0.05)
	3	0.23 aB (0.03)	0.23 aB (0.06)	0.32 aC (0.05)	0.41 aC (0.14)
	6	0.34 aC (0.02)	0.54 bC (0.13)	0.29 aC (0.08)	0.30 aBC (0.07)

* Average values of four (*n* = 4) replicates; standard deviations are indicated in brackets. In each column, different capital letters (A–C) indicate significant differences (*p* < 0.05) as a result of previous frozen storage time. In each row, different lowercase letters (a, b) indicate significant differences (*p* < 0.05) as a result of OCJ concentration. ** Abbreviations: PV (peroxide value), TBA-i (thiobarbituric acid index), and OCJ (octopus cooking juice). Packing systems: CT (control), OCJ-1 (low-concentrated OCJ), OCJ-2 (medium-concentrated OCJ), and OCJ-3 (high-concentrated OCJ) batches, according to preparation described in the Materials and Methods section.

**Table 2:** Determination * of fluorescence ratio (FR) and polyene index (PI) in initial and canned horse mackerel previously subjected to frozen storage and canned with different packing systems **.

	Storage Time (Months)	Packing System			
		**CT**	**OCJ-1**	**OCJ-2**	**OCJ-3**
**FR**	Initial	1.36 A (0.41)	1.36 A (0.41)	1.36 A (0.41)	1.36 A (0.41)
	0	4.43 aB (0.54)	4.06 aB (0.27)	4.13 aB (0.49)	3.99 aB (0.19)
	3	5.32 bC (0.17)	4.53 aB (0.30)	4.23 aB (0.38)	4.31 aB (0.49)
	6	5.93 bC (0.58)	4.76 abB (0.95)	4.21 abB (0.74)	3.92 aB (0.26)
					
**PI**	Initial	1.42 B (0.12)	1.42 B (0.12)	1.42 B (0.12)	1.42 B (0.12)
	0	1.22 aB (0.11)	1.18 aA (0.15)	1.41 aB (0.32)	1.23 aAB (0.09)
	3	1.10 aA (0.05)	1.38 aAB (0.15)	1.19 aAB (0.18)	1.18 aA (0.06)
	6	0.98 aA (0.13)	1.12 aA (0.14)	1.14 aA (0.09)	1.28 aAB (0.18)

* Average values of four (*n* = 4) replicates; standard deviations are indicated in brackets. In each column, different capital letters (A–C) indicate significant differences (*p* < 0.05) as a result of previous frozen storage time. In each row, different lowercase letters (a, b) indicate significant differences (*p* < 0.05) as a result of OCJ concentration. ** Abbreviations: OCJ (octopus cooking juice). Packing systems as expressed in Table 1.

**Table 3:** Determination * of colour values in initial and canned horse mackerel previously subjected to frozen storage and canned with different packing systems **.

	Storage Time (Months)	Packing System			
		CT	OCJ-1	OCJ-2	OCJ-3
L*	Initial	44.04 A (3.49)	44.04 A (3.49)	44.04 A (3.49)	44.04 A (3.49)
	0	67.66 cB (1.50)	60.01 bAB (0.71)	60.51 bB (0.73)	54.31 aB (1.49)
	3	66.14 cB (0.56)	65.34 cC (1.25)	59.63 bB (1.05)	57.07 aC (0.82)
	6	70.15 cC (0.52)	66.50 bC (2.60)	63.46 abC (1.77)	61.16 aD (1.25)
					
B*	Initial	3.33 A (0.04)	3.33 A (0.04)	3.33 A (0.04)	3.33 A (0.04)
	0	14.08 aB (1.84)	14.37 aB (0.75)	14.86 aB (1.62)	13.17 aB (0.60)
	3	17.05 bB (1.84)	16.34 bB (1.25)	15.63 bB (1.16)	12.97 aB (1.23)
	6	18.47 cC (0.26)	18.45 cC (0.53)	16.25 bB (0.78)	14.41 aB (0.61)

* Average values of four (*n* = 4) replicates; standard deviations are indicated in brackets. In each column, different capital letters (A–D) indicate significant differences (*p* < 0.05) as a result of previous frozen storage time. In each row, different lowercase letters (a–c) indicate significant differences (*p* < 0.05) as a result of OCJ concentration. ** OCJ (octopus cooking juice). Packing systems as expressed in Table 1.

## Data Availability

The data are contained within the article.
